# Filling in the Gap of Human Chromosome 4: Single Molecule Real Time Sequencing of Macrosatellite Repeats in the Facioscapulohumeral Muscular Dystrophy Locus

**DOI:** 10.1371/journal.pone.0151963

**Published:** 2016-03-22

**Authors:** Masaki Suimye Morioka, Miwako Kitazume, Ken Osaki, Jonathan Wood, Yujiro Tanaka

**Affiliations:** 1 Dept. of Bioinformatics, Medical Research Institute, Tokyo Medical and Dental University, 1-5-45 Yushima, Bunkyoku, Tokyo, 113–8510, Japan; 2 Tomy Digital Biology, SF Build. 7F, 1-4-6 Nezu, Bunkyoku, Tokyo, 113–0031, Japan; 3 Genome Reference Informatics T135, Genome Reference Consortium, Wellcome Trust Sanger Institute, Wellcome Trust Genome Campus, Hinxton, Cambridge, CB10 1SA, United Kingdom; 4 Genome Structure and Regulation, Graduate School of Biomedical Science and Dept. of Biochemical Genetics, Medical Research Institute, Tokyo Medical and Dental University, 2-3-10 Kandasurugadai, Chiyodaku, Tokyo, 101–0062, Japan; University of Minnesota, UNITED STATES

## Abstract

A majority of facioscapulohumeral muscular dystrophy (FSHD) is caused by contraction of macrosatellite repeats called D4Z4 that are located in the subtelomeric region of human chromosome 4q35. Sequencing the FSHD locus has been technically challenging due to its long size and nearly identical nature of repeat elements. Here we report sequencing and partial assembly of a BAC clone carrying an entire FSHD locus by a single molecule real time (SMRT) sequencing technology which could produce long reads up to about 18 kb containing D4Z4 repeats. De novo assembly by Hierarchical Genome Assembly Process 1 (HGAP.1) yielded a contig of 41 kb containing all but a part of the most distal D4Z4 element. The validity of the sequence model was confirmed by an independent approach employing anchored multiple sequence alignment by Kalign using reads containing unique flanking sequences. Our data will provide a basis for further optimization of sequencing and assembly conditions of D4Z4.

## Introduction

**Fig 1:**
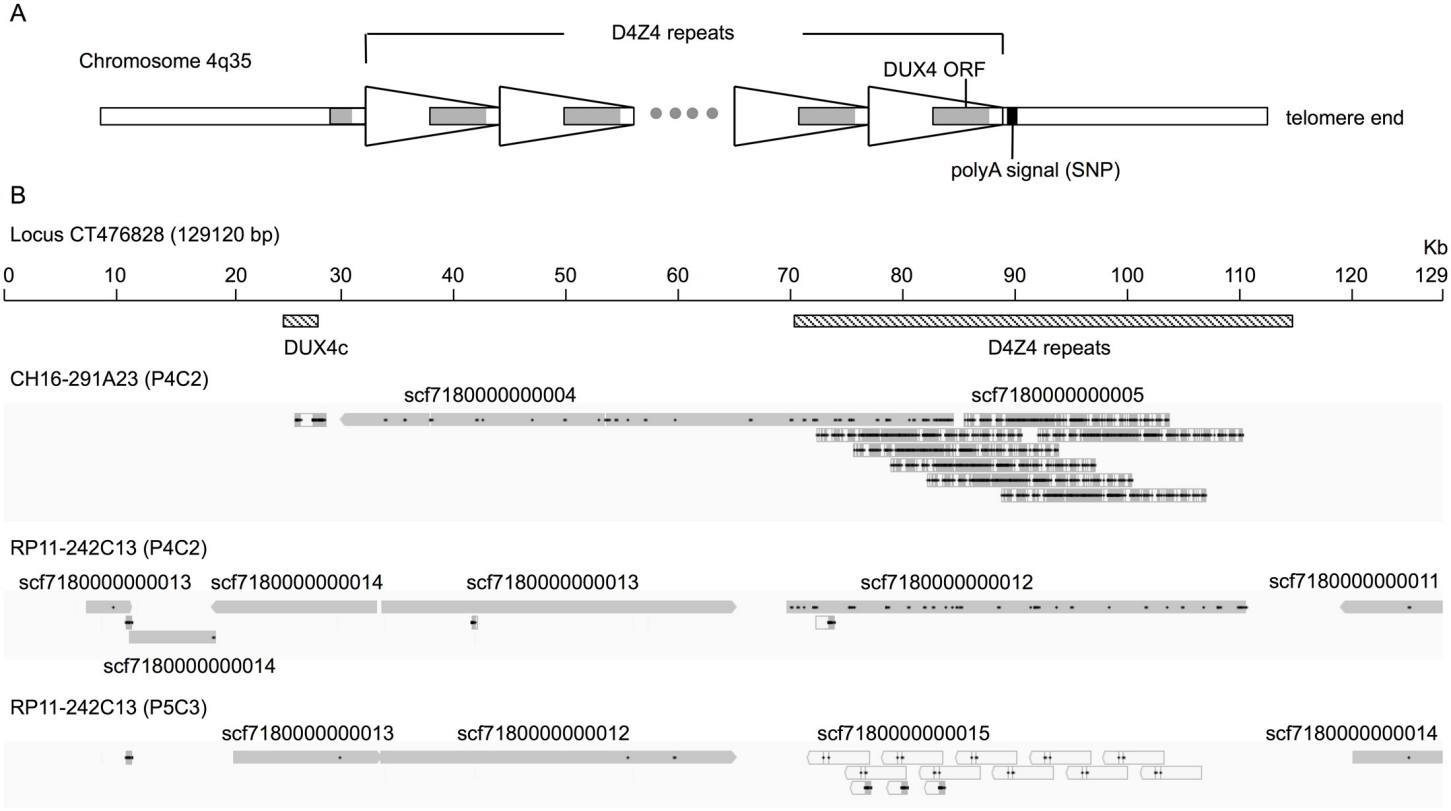
Elements of the FSHD locus and positions of polished contigs of BAC clones. A. Organization of the FSHD locus of the human chromosome 4 subtelomeric region. The D4Z4 repeat unit is represented by trapezoid with an inner shaded rectangle of the DUX4 coding region. The copy number of D4Z4 that causes the disease depends on the genetic background of secondary modifiers such as SMCHD1. B. Alignments of polished contigs of CH16-291A23 (P4C2), RP11-242C23 (P4C2), and RP11-242C23 (P5C3) to a reference, i.e. Genebank accession number CT476828 that comprises 13 copies of an identical D4Z4 unit. Contig names are indicated above grey boxes, where black dots represent gaps. White boxes are contigs of low quality scores. For CH16-291A23 (P4C2) and RP11-242C23 (P5C3), contigs containing D4Z4 units are mapped multiple times to the D4Z4 repeat region since they cannot be uniquely mapped. Note that scf180000000014 has not been correctly assembled and consists of two fragments, one of which contains an inverted D4Z4 unit (DUX4c).

About half [1] and possibly more [2] of the human genome comprise repetitive elements. Macrosatellite repeats, consisting of units longer than 100 bp and often spanning several kilobases (kb), have attracted attention in recent years for their possible structural and regulatory roles [3]. In the subtelomeric region of human chromosome 4 are tandem arrays of 3.3 kb macrosatellite repeats called D4Z4. The D4Z4 repeats are highly polymorphic among healthy individuals ranging from 8 to 100 copies (Fig 1A), whereas a majority of FSHD patients have less than eleven but more than one copies of D4Z4 [4–7]. Contraction of these repeats is thought to cause relaxation of the chromatin structure, leading to derepression and transcriptional activation of the locus by a Trithorax-group protein ASH1 [8–10]. A SNP located at the distal end of the D4Z4 repeats is a transcription termination signal AATAAA in approximately half of the population, and when combined with short D4Z4 repeats results in the synthesis of DUX4 proteins encoded in the most distal D4Z4 unit [11]. The copy number of D4Z4 that manifests the disease also depends on secondary modifiers such as SMCHD1, mutation of which causes derepression of loci containing more than 10 copies of D4Z4 [12].

Nearly identical long repeats such as D4Z4 cannot be assembled by aligning sequence reads shorter than the repeat length. In addition, D4Z4 has been hard to sequence due to its high GC content [5–6]. The SMRT sequence technology could be a solution to such problems, since it provides reads longer than several kb and the Phi29 DNA polymerase used is highly robust against GC-rich tracts [13–14]. On the other hand, the SMRT sequence is prone to insertion/deletion type errors that can be up to 15%, far exceeding sequence variations among D4Z4 repeats. The SMRT sequence errors, however, are random in nature and thus could be corrected if provided with sufficient coverage and accurate mapping.

The latest human genome assembly GRCh38.p5 released on September 15, 2015 (S1 Fig, http://www.ncbi.nlm.nih.gov/projects/genome/assembly/grc/human/) still contains 14 open gaps including one in the chromosome 4. Here we report sequencing and partial assembly of a BAC clone spanning an entire D4Z4 repeat region of the human chromosome 4.

## Materials and Methods

### BAC clones

CH16-291A23 and RP11-242C23 human BAC clones were obtained from BAC PAC Resources Center in Children's Hospital Oakland, USA. CHORI-16 and RPCI-11 BAC libraries were constructed from randomly chosen male donors [15]. CH16-291A23 was identified by Cabianca et al. [10] and RP11-242C23 was sequenced and partially assembled by Genome Reference Consortium, Wellcome Trust Sanger Institute (GenBank Accession Number CT476828.7).

### SMRT sequencing

BAC DNA was first purified by MoBio PowerClean Kit, sheared by centrifugation through g-Tube, and then concentrated by AMpure. SMRT bell libraries were constructed using DNA Template Prep Kit v2.0 and then analyzed by PacBio RS using DNA/Polymerase Binding Kit P4, Sequencing reagent v2.0, and a single SMRT cell v3 (P4C2) [13–14]. Additionally, RP11-242C23 was analyzed by PacBio RS using DNA/Polymerase Binding Kit P5 and Sequencing reagent v3 (P5C3). Sequenced read qualities and GC contents of D4Z4 repeats and other regions were evaluated by using FastQC program version 0.11.2 (http://www.bioinformatics.babraham.ac.uk/projects/fastqc/).

### De novo assembly by HGAP pipeline

HGAP.1 pipeline was employed for de novo assembly of CH16-242C23 and RP11-242C23 SMRT sequence data each from a single cell. Briefly, sequence errors were first corrected by mapping short subreads (< 500 bp) to longer subreads (> 500 bp) using Basic Local Alignment with Successive Refinement (BLASR), and then assembled by Celera Assembler v.7.0 to obtain contigs [13–14]. To further polish contigs, error corrected subreads were remapped to the contigs. The HGAP.3 pipeline was used for resequencing the RP11-242C23 P5C3 data to the longest filtered subreads containing the 5' or 3' unique flanking sequences.

### Multiple sequence alignment by Kalign

A query sequence containing entire D4Z4 repeats and both upstream and downstream 500 bp unique flanking sequences was derived from the published RP11-242C23 sequence (CT476828.7). NCBI blastn [16] was used to extract sequences that aligned to the query (word size 50, percent identity 85). The resulting sequences were further selected by the presence of either 5' or 3' flanking sequences using blastn. Subsequently Kalign version 1.04 algorithm [17] implemented in Unipro UGENE environment [18] was used to produce multiple sequence alignment. A consensus was built by accepting bases above threshold values, i.e. 13% for P4C2 data and 14% for P5C3 data as determined by visual inspection of alignments towards the 3' end where signal/noise ratios tend to become lower. Identities and differences between contigs and consensus sequences were calculated by CLC Main Workbench Version 6.9 (https://www.qiagenbioinformatics.com/) Create Pairwise Comparison. Dot plot representation of sequence similarities was generated by Create Dotplot program in CLC Main Workbench.

### Clustering of Sanger reads

Sanger sequences of RP11-242C23 were obtained from the NCBI Trace Archive (http://www.ncbi.nlm.nih.gov/Traces/trace.cgi) by searching center_project = 'bA242C23' and center_name = 'SC'. Five hundred reads containing D4Z4 were identified by NCIB blastn and arranged in the order of their 5' ends, grouped into four overlapping subsets (1–200, 101–300, 201–400, 301–500), and clustered by CLC Main Workbench Create Alignment algorithm with Gap open cost 10.0 and Gap extension cost 1.0. CLC Main Workbench Create Tree was used to generate trees with Neighbor Joining algorithm, Jukes-Kantor distance, and 1000 bootstrap analysis.

## Results

### Sequencing results of a BAC clone CH16-291A23

To test the feasibility of SMRT technology for sequencing D4Z4 repeats, we first analyzed a BAC clone CH16-291A23 that contains the proximal D4Z4 repeat region [10]. For library construction, CH16-291A23 was subjected to random fragmentation by g-Tube and then concentrated by AMpure. Subsequently, DNA fragments were processed with P4C2 chemistry and sequenced using a single cell of PacBio RS according to the manufacture's protocol [13–14]. As summarized in Table 1, 66,246 filtered subreads and total of 286,932,352 bases were obtained. Mean and maximum read length were 4,331 and 22,039 bases, respectively.

**Table 1:** Statistics of filtered subreads. Statistics of filtered subreads of the two BAC clones. Numbers of filtered subreads and quality scores are similar for three samples.

BAC clone	Chemistry	Number of filtered Reads	Number of Bases	Mean Read Length	Mean Filtered read Score	N50 Read Length	Maximum filtered read length
CH16- 291A23	P4C2	66246	286932352	4331	0.850	6377	22039
RP11- 242C23	P4C2	71180	452185788	4534	0.840	8340	32845
RP11- 242C23	P5C3	64451	553113108	4539	0.838	10266	32152

De novo assembly of the filtered subreads of CH16-291A23 by HGAP.1 produced two contigs, longer one of which was 85,677 bases (Table 2). Both long and short contigs contained ten complete and one partial D4Z4 elements in total as depicted in Fig 1B. The distal end of the D4Z4 repeat region was absent from this BAC clone as predicted. A 100% accuracy of the vector sequence supported the validity of this assembly.

**Table 2:** Statistics of contigs. Basic statistics of contigs generated from two BAC clones and two kinds of library construction chemistries. CH16-291A23 and RP11-242C23 with P4C2 chemistry generated contigs containing D4Z4 repeats. RP11-242C23 prepared by P5C3 chemistry failed to produce contigs with D4Z4 repeats.

	Chemistry	Polished contigs	Max contig length	Sum of contig lengths
CH16-291A23	P4C2	2	85677	103630
RP11-242C23	P4C2	5	41097	110468
RP11-242C23	P5C3	4	31377	60813

### Sequencing results of BAC clone RP11-242C23

The BAC clone RP11-242C23 has been recently sequenced and partially assembled by Genome Reference Consortium, Wellcome Trust Sanger Institute. The clone spans an entire FSHD locus but the D4Z4 sequences deposited in the database are not experimentally verified sequences but instead consist of exact copies of a single representative D4Z4 element. As summarized in S1 Table and S2 Fig, digestion of RP11-242C23 with several restriction enzymes revealed minimal length of the repeat region of about 48 kb, corresponding roughly to 13.5 copies of D4Z4 together with 5' and 3' flanking sequences. Sanger reads are about 1 kb in length and 500 sequences containing D4Z4 were identified by NCBI blastn search. These were arranged in the order of the 5' ends, grouped into four overlapping subsets, and subjected to clustering analysis. Two or three clusters (instead of 13 or 14) were clearly visible in each subset (S3 Fig), but it was not obvious how these clusters relate to each one of the predicted 13.5 D4Z4 repeats.

**Fig 2:**
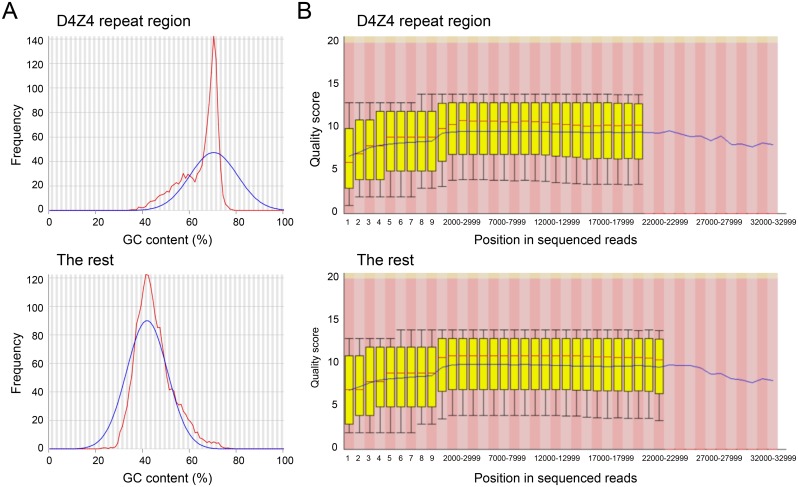
GC contents and read qualities of D4Z4 repeat region and the rest. A. GC contents of D4Z4 repeats and the rest of the BAC clone RP11-242C23 (P4C2 chemistry). Red lines indicate GC counts per read and blue lines indicate theoretically modeled normal distributions. D4Z4 repeats are highly rich in GC. B. Box plots showing the predicted per-base quality scores of sequenced reads at various positions from 5' to 3' ends for D4Z4 repeats and the rest.

To sequence RP11-242C23 by the SMRT technology, DNA was first digested with EcoRV to isolate the D4Z4 region and further fragmented by g-Tube before library construction by either P4C2 or P5C3 chemistry. The P5C3 chemistry is optimized for obtaining longer reads (S4 Fig). As summarized in Table 2, the SMRT sequence produced 71,180 and 64,451 filtered subreads by P4C2 and P5C3 chemistries, respectively. Sequencing qualities were similar in P4C2 and P5C3 reads as indicated by mean filtered read scores (Table 1). Of note, quality scores across various read positions were similar between the D4Z4 region and the rest (Fig 2B) despite the fact that D4Z4 region has higher GC content (70%) than that of the rest (Fig 2A). These data suggest that the high GC content of D4Z4 repeats had little impact on sequencing qualities.

**Fig 3:**
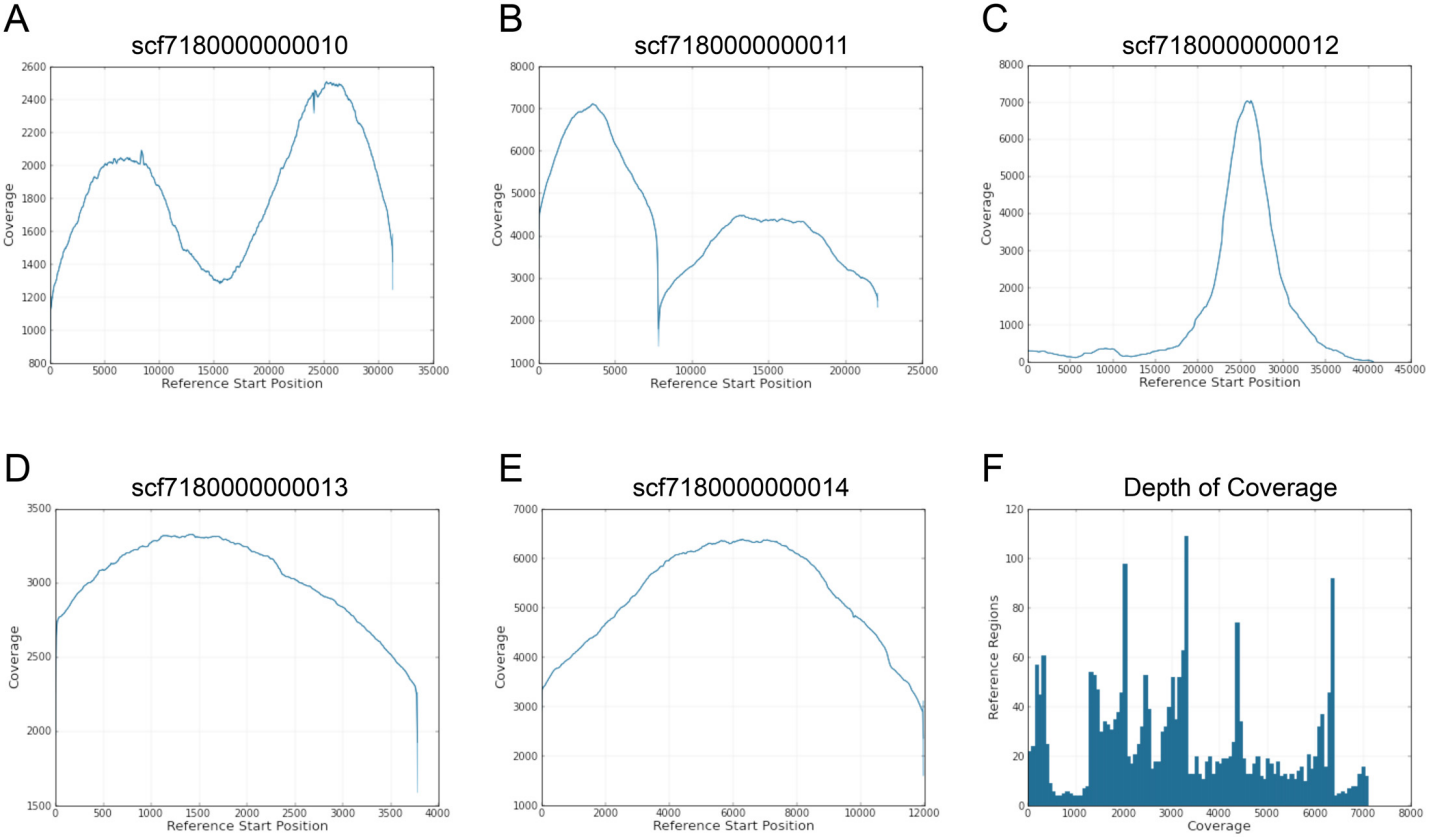
Coverage of RP11-242C23 sequencing by P4C2 chemistry. A-E. Horizontal axis is the positon in each contig and the vertical axis is the coverage for the five contigs generated by HGAP.1 analysis of RP11-242C23 (P4C2). Contigs that can be mapped to a reference, i.e. Genebank accession number CT476828, are illustrated in Fig 1B. Sharp drops correspond to EcoRV sites. The contig containing D4Z4 repeats (C) shows highly skewed coverage distribution, suggesting misalignment in the middle region. F. Frequencies of different levels (depth) of coverage are shown.

The HGAP.1 pipeline employed to assemble P4C2 and P5C3 reads of RP11-242C23 generated five and four contigs, respectively, as summarized in Table 2 and S2 Table. The longest contig of P4C2 was 41,097 bases and covered all D4Z4 repeats except for a part of the most distal D4Z4 as illustrated in Fig 1B. By contrast, the longest contig of P5C3, which was 31,377 bases in length, did not contain D4Z4 repeats despite the fact that there was little difference in sequence qualities between P4C2 and P5C3 data as noted above. Fig 3 shows coverages of the five contigs of P4C2, where scf7180000000012 is the one containing D4Z4. The coverage of this contig is highly skewed in the middle region, suggesting errors in mapping repetitive sequences. By contrast, approximately 20 kb in the 5' part of scf7180000000012 shows relatively even coverage, suggesting reads were mapped correctly in this region. Also of note is that de novo assembly of P4C2 subreads, i.e. the contig scf7180000000014 of RP11-242C23 (P4C2) in Fig 1B, failed to properly assemble DUX4c, an inverted copy of D4Z4 37 kb upstream of the D4Z4 repeats, suggesting that D4Z4 sequences from the two regions might have caused mapping errors.

**Fig 4:**
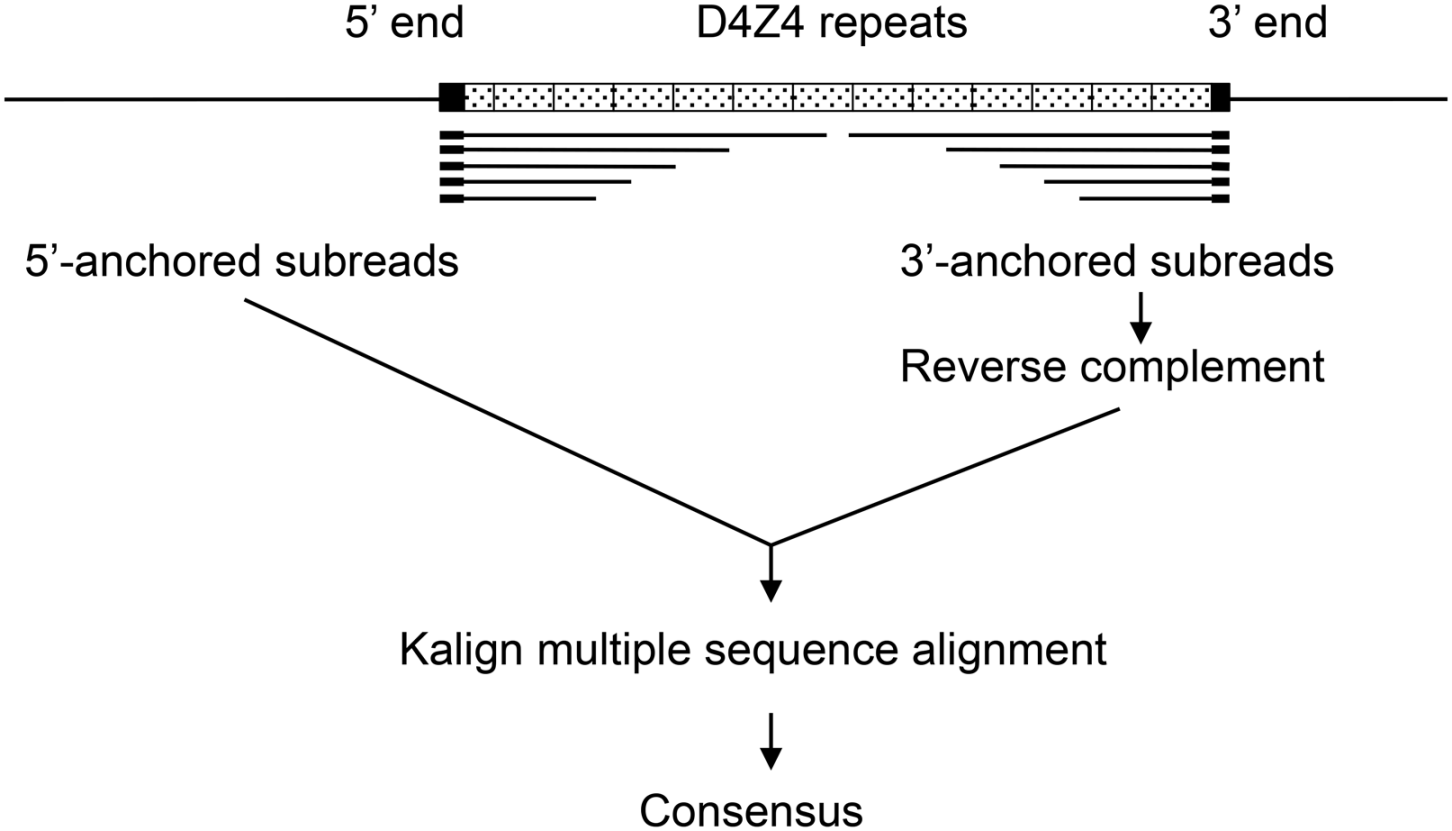
Scheme of anchored multiple sequence alignment. To avoid misalignment of repetitive elements, quality-filtered subreads containing D4Z4 and flanking unique sequences were first selected and trimmed by either 5' or 3' unique region. Sequences at the 3' end were reverse complemented to bring unique sequences to the 5' end. After generating multiple sequence alignment by Kalign, consensus sequences were generated by calling bases above thresholds (13% for P4C2, 14% for P5C3).

To verify the accuracy of the P4C2 contig model by an independent method, Kalign multiple sequence alignment algorithm [17] was employed to align sequences that contain either 5' or 3' flanking unique sequences in addition to various lengths of D4Z4 repeats. As depicted in Fig 4, flanking sequences serve anchors to help align repetitive sequences at the right position. Subsequently, bases above thresholds were called to generate consensus sequences. As shown in Table 3, reads as long as 18,423 and 18,971 bases from the 5' end (NDE; non-deleted element) and the 3' end (pLAM), respectively, of the D4Z4 repeat region were extracted from P5C3 data.

**Table 3:** Statistics of anchored subreads. Filtered subreads of P4C2 and P5C3 data were selected and trimmed by 5' or 3' flanking sequences. Those containing the unique NDE or pLAM elements were subjected to multiple sequence alignment by Kalign. The longest subread of P5C3 corresponds to about 5.5 copies of D4Z4.

	P4C2		P5C3	
	Max coverage	Max length	Max coverage	Max length
Reads with 5' NDE	177	16036	147	18423
Reads with 3' pLAM	112	11813	130	18971

P5C3 data generated consensus sequences containing 3.5 and 4 copies of D4Z4 from the 5' and 3' ends respectively (S5 Fig). P4C2 data had slightly shorter reads covering the D4Z4 region, and produced consensus sequences containing only two copies each from the 5' and 3' ends (S5 Fig). Comparison of D4Z4 repeat units 1, 12, and 13 (Table 4) shows that sequences obtained by different methods and different samples are highly similar to each other, and HGAP.1 de novo assembly of P4C2 and Kalign consensus of P5C3 shared more than 99% identity at D4Z4 1 and 12.

**Table 4:** Pair-wise comparisons of D4Z4 sequences obtained by independent methods. Pair-wise comparisons between P4C2-Kalign, P5C3-Kalign, and P4C2-HGAP.1 were carried out by CLC Main Workbench (version 6). Percent identities and number of nucleotide differences are shown.

	D4Z4_1		D4Z4_12		D4Z4_13	
Comparison	Percent identity	Difference	Percent identity	Difference	Percent identity	Difference
P4C2:P5C3	97.40	87	98.91	36	98.98	34
HGAP.1:P4C2	97.49	84	98.97	34		
HGAP.1:P5C3	99.85	5	99.28	24		

**Fig 5:**
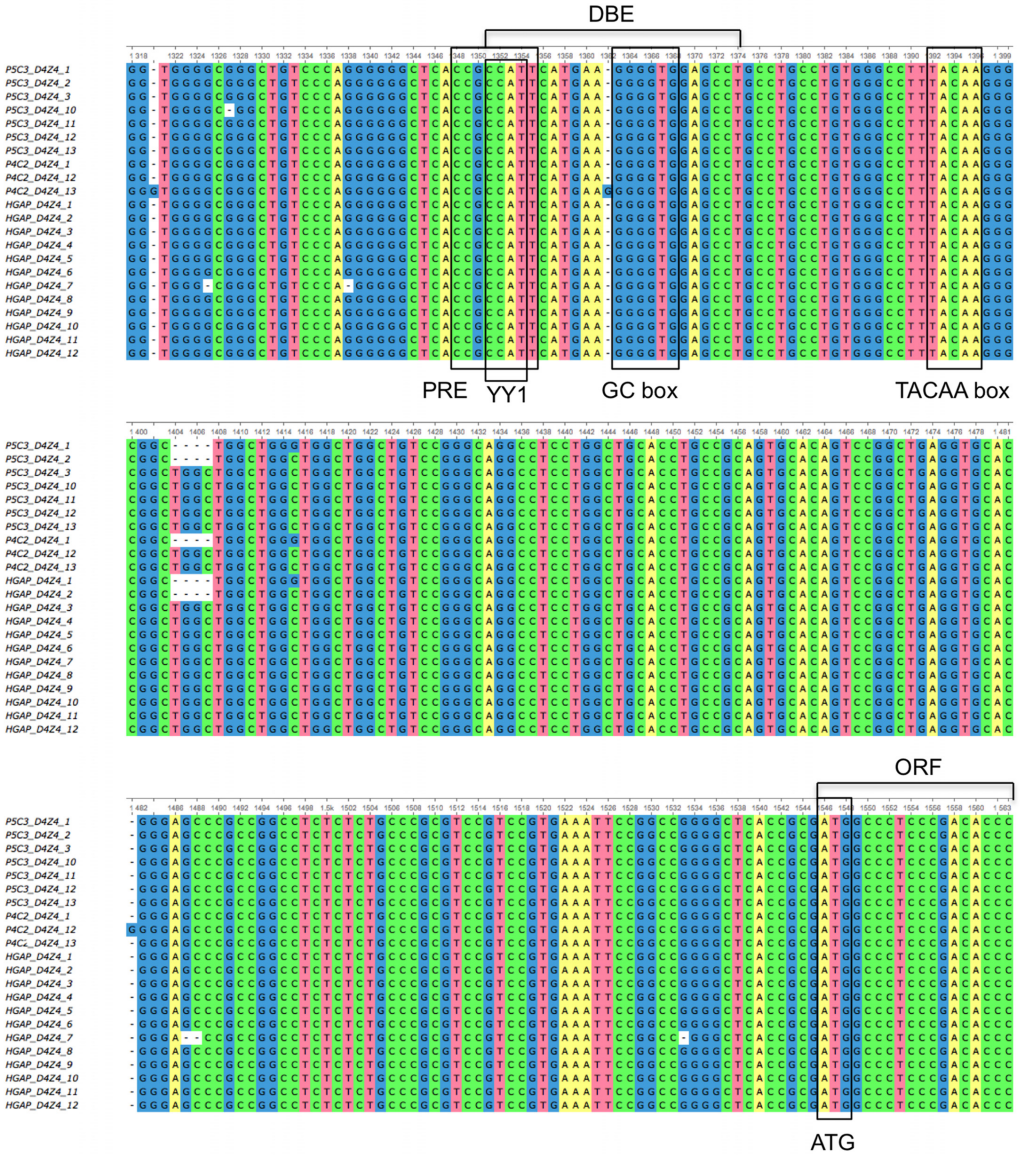
Conservation of transcription factor binding motifs in the D4Z4 array. A total of D4Z4 sequences obtained by either anchored multiple sequence alignment (P5C3_ and P4C2_ followed by positions in the array) or resequencing using longest P5C3 subreads containing D4Z4 and 5' or 3' flanking unique sequences (HGAP_ followed by positions in the array) were aligned to each other to assess sequence variations. Depicted are regions upstream of ATG translation start site that contain known motifs such as PRE, YY1, GC box, and TACAA box.

To further assess sequence variations of D4Z4 in RP11-242C23, a total of 22 D4Z4 sequences that were obtained by either anchored multiple sequence alignment or resequencing using longest P5C3 subreads containing D4Z4 and either 5' or 3' unique regions were compared to each other (S6 and S7 Figs). Fig 5 highlights conservation of known transcriptional regulatory motifs in the region upstream of the ATG codon including PRE, YY1, GC box, and TACAA box. All D4Z4 units share identical sequence motifs in this region, suggesting that D4Z4 units in this array are regulated by the same mechanisms.

## Discussion

Despite progresses in sequencing technologies in recent years, GC-rich and long repetitive elements such as D4Z4 still pose a technical challenge. The fundamental problem is the difficulty in accurately mapping reads to each other or to references containing repetitive elements. Even long reads of more than several kb produced by the SMRT sequence technology of PacBio RS cannot be precisely mapped if not provided with anchors that can be unambiguously placed. Thus, a general strategy of HGAP, an error correction of long reads by short accurate reads, does not work well with D4Z4 repeats.

The SMRT sequence technology was recently employed to aid assemble a haploid human genome of hydatidiform mole CHM1 [19–20]. Although SMRT reads closed 50 gaps and extended 60 boundaries out of 160 gaps remaining in the human reference genome GRCh37, the chromosome 4 subtelomeric gap was not closed because highly identical repeats could not be resolved. In the current study, we sequenced a BAC clone by P4C2 and P5C3 chemistries. Unexpectedly, de novo assembly of only the P4C2 data generated a contig that contained the D4Z4 repeat region, despite the fact that P5C3 reads had similar sequence qualities as P4C2 reads. By contrast, an independent approach of anchored multiple sequence alignment using unique sequences flanking the D4Z4 repeats for precise alignment revealed that P5C3 data had more and longer reads containing the anchor sequences and as a consequence produced longer consensus sequences. Comparisons between de novo assembly by HGAP.1 and anchored multiple sequence alignment showed that consensus sequences obtained by independent methods were highly similar to each other. These data demonstrate the validity of the D4Z4 sequence model except for the middle part that could not be reached by the current SMRT sequence technology. A small number of sequence errors also remained in the D4Z4 sequence model and these have to be resolved in the future either by increasing the depth of coverage or by extending the read length.

To understand molecular epigenetic mechanisms underlying FSHD, it is important to know how binding of chromatin regulatory factors such as ASH1 to the FSHD locus is regulated in healthy individuals and how they are altered in FSHD patients [9, 21]. Accurate sequences and the information about sequence variations among D4Z4 copies could facilitate molecular studies of the locus at the level of each repeat unit, which in turn would be important for developing targeted therapeutic measures to ameliorate the disease.

As to the assessment of copy numbers of D4Z4 repeats, the current SMRT sequence technology can already produce reads longer than 30 kb. Importantly, P5C3 data contained reads longer than 18 kb from the 5' and 3' edges of D4Z4 repeats, 5 each covering 5 copies of D4Z4. Since a majority of FSHD patients carry less than eleven copies of D4Z4 [8], the SMRT sequence could become a feasible method for genetic diagnosis of FSHD in the future. To assist such an approach, we are currently developing a way to enrich DNA fragments containing the D4Z4 region from genomic DNA.

## Supporting Information

S1 Fig Human reference genome in chromosome 4q35 subtelomeric region. (TIF)

S2 Fig Pulse Field Gel Electrophoresis Analysis of RP11-242C23. (TIF)

S3 Fig Clustering analysis of Sanger reads of RP11-242C23. (TIF)

S4 Fig Read length distribution of RP11-242C23 sequencing by P4C2 or P5C3 chemistries. (TIF)

S5 Fig Dot plot representation of pair-wise alignment of D4Z4 consensus sequences. (TIF)

S6 Fig The 5' half of aligned consensus sequences of D4Z4. (TIF)

S7 Fig The 3' half of aligned consensus sequences of D4Z4. (TIF)

S1 Table Estimated DNA fragment sizes of RP11-242C23 digested with restriction endonucleases. (XLSX)

S2 Table Coordinates of de novo assembly contigs of RP11-242C23. (XLSX)

S1 Data A compressed file containing all sequence data. (ZIP)
