# Supplementary figures and images for: Filling in the Gap of Human Chromosome 4: Single Molecule Real Time Sequencing of Macrosatellite Repeats in the Facioscapulohumeral Muscular Dystrophy Locus
Source: PLoS One. 2016 Mar 22;11(3):e0151963. doi: 10.1371/journal.pone.0151963 (PMC4803325; doi:10.1371/journal.pone.0151963)

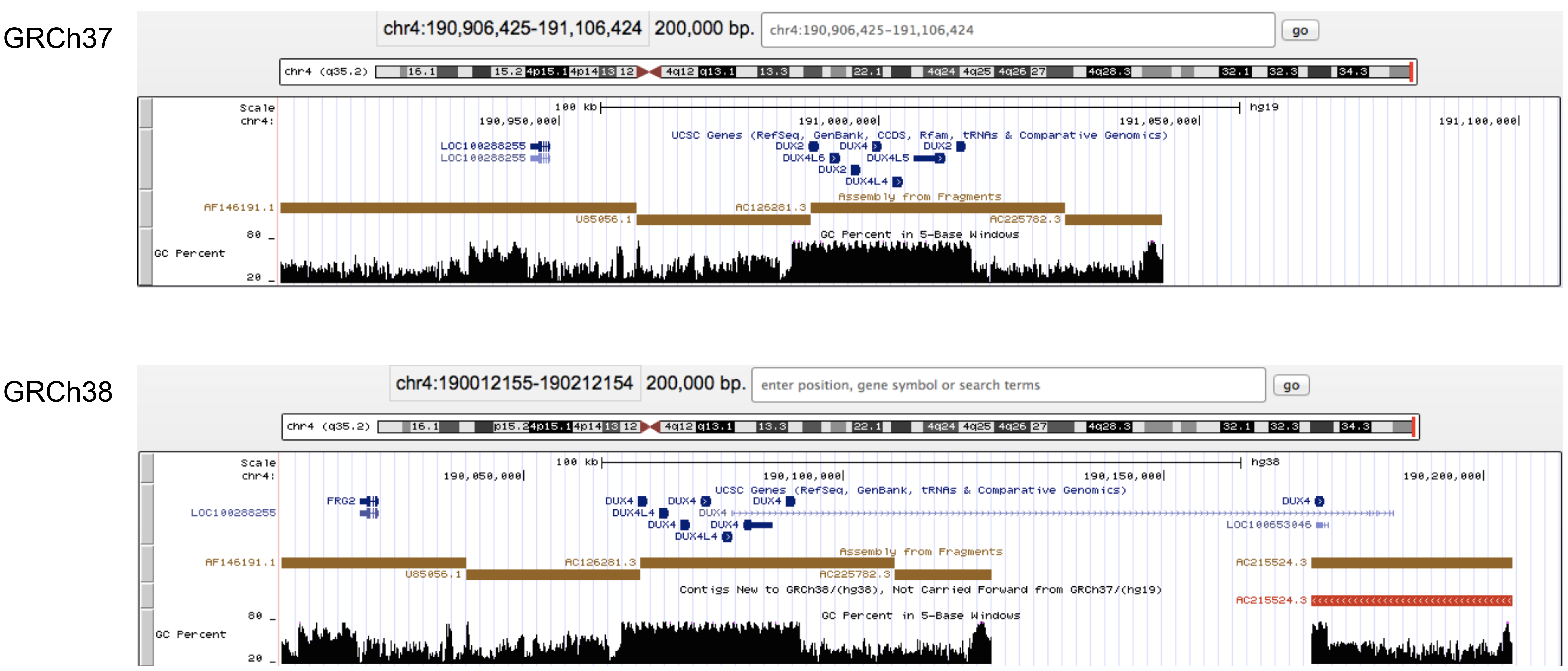

Supplement: S1 Fig — (TIF) [file pone.0151963.s001.tif]

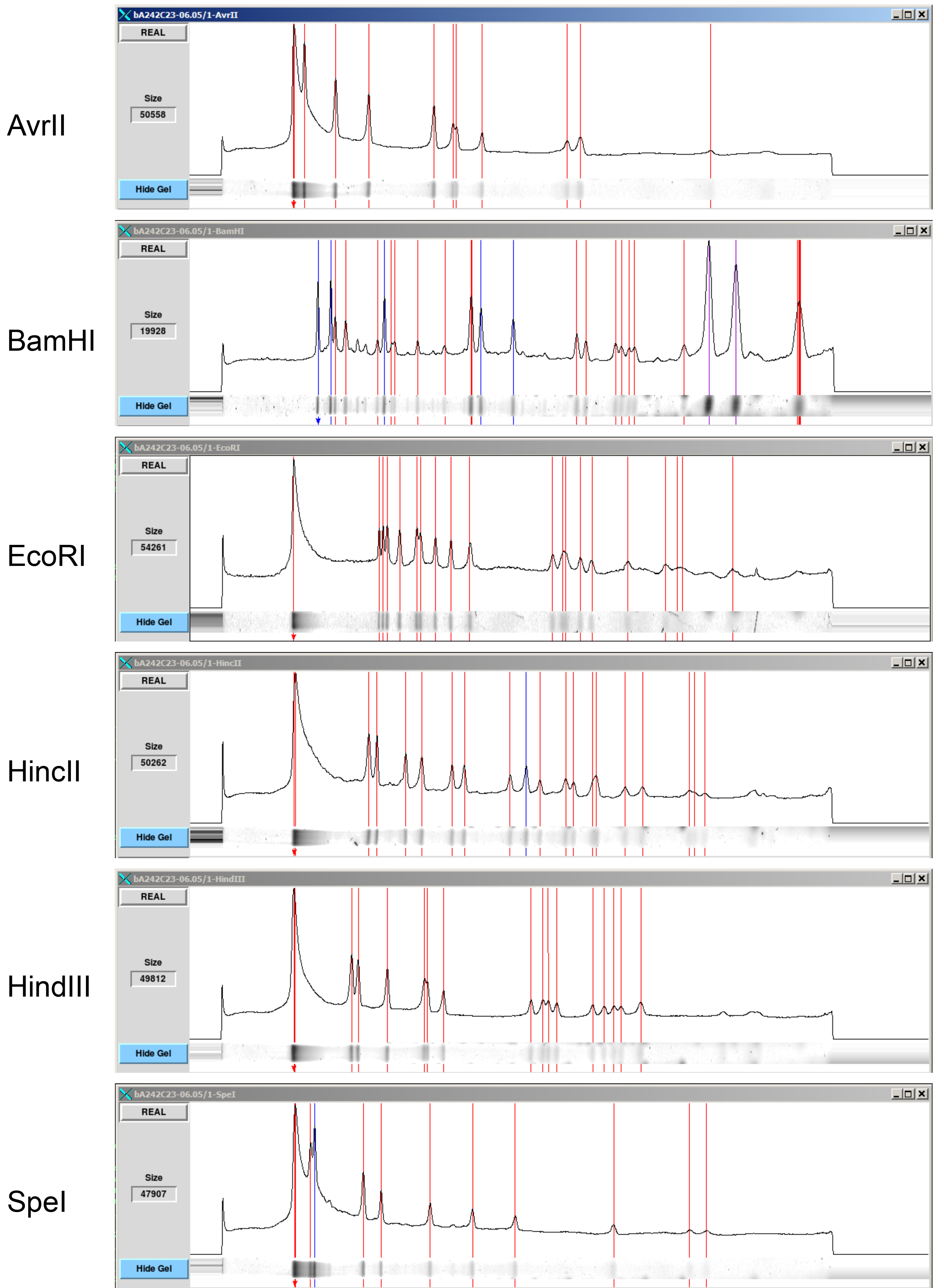

Supplement: S2 Fig — (TIF) [file pone.0151963.s002.tif]

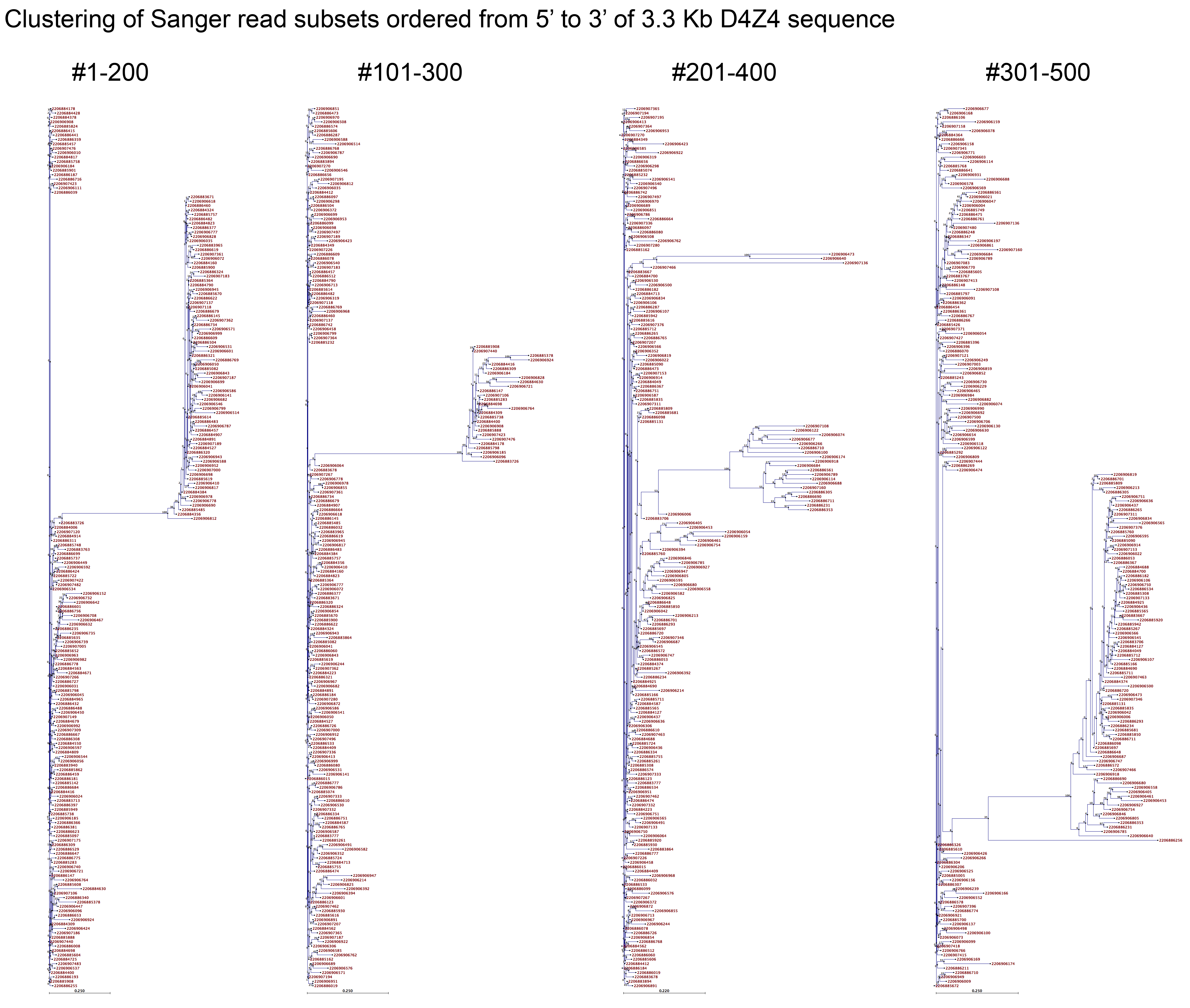

Supplement: S3 Fig — (TIF) [file pone.0151963.s003.tif]

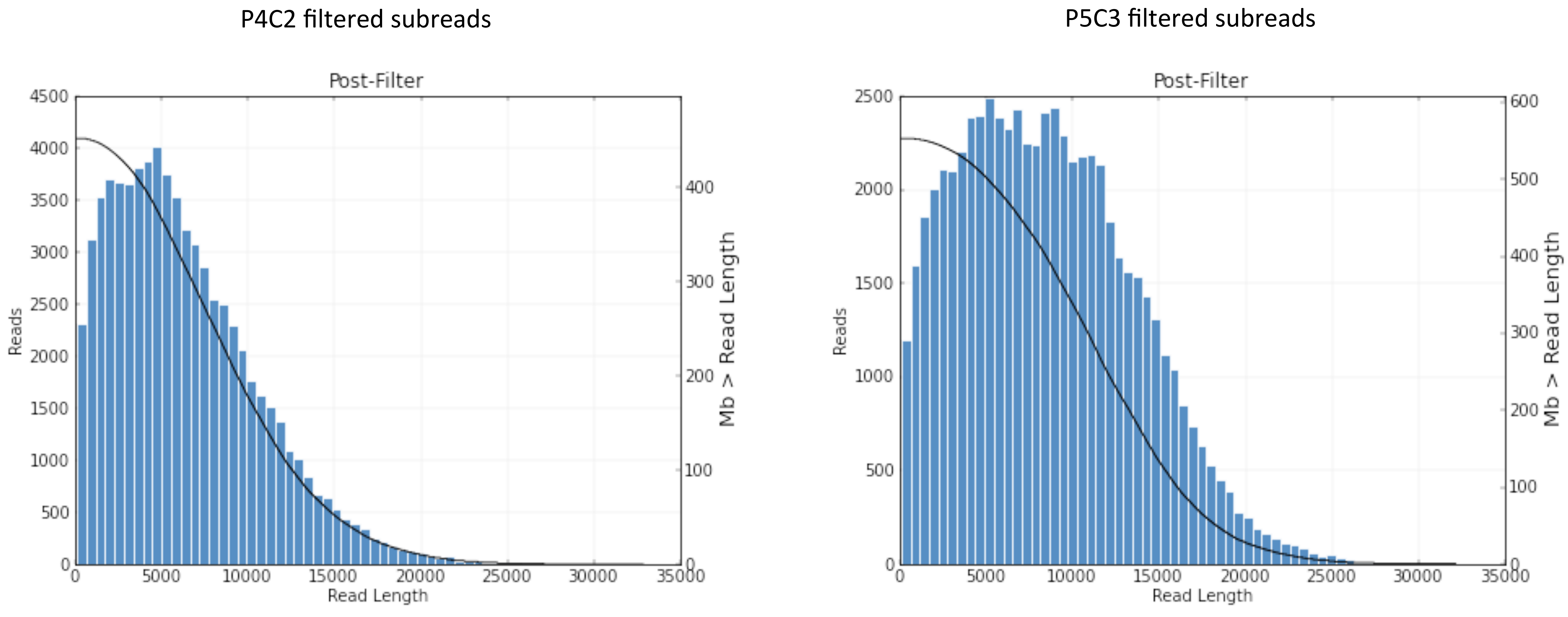

Supplement: S4 Fig — (TIF) [file pone.0151963.s004.tif]

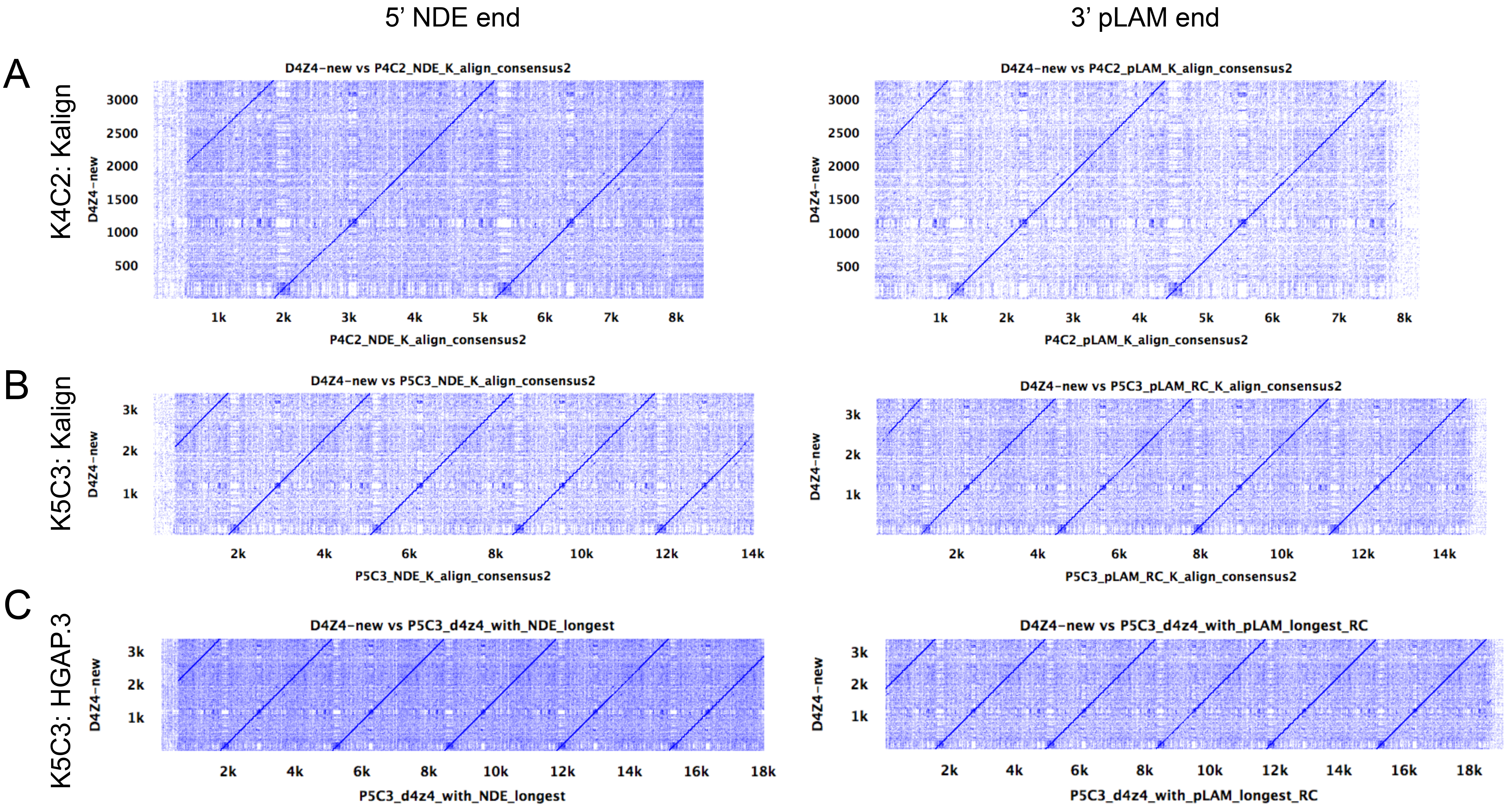

Supplement: S5 Fig — (TIF) [file pone.0151963.s005.tif]
